# Condition Monitoring of Railway Crossing Geometry via Measured and Simulated Track Responses

**DOI:** 10.3390/s22031012

**Published:** 2022-01-28

**Authors:** Marko D. G. Milosevic, Björn A. Pålsson, Arne Nissen, Jens C. O. Nielsen, Håkan Johansson

**Affiliations:** 1 Department of Mechanics and Maritime Sciences/CHARMEC, Chalmers University of Technology, 412 96 Gothenburg, Sweden; bjorn.palsson@chalmers.se (B.A.P.); jens.nielsen@chalmers.se (J.C.O.N.); Hakan.Johansson@chalmers.se (H.J.); 2 The Swedish Transport Administration, P.O. Box 809, 971 25 Luleå, Sweden; arne.nissen@trafikverket.se

**Keywords:** condition monitoring, railway, crossing geometry, accelerometer, 3D scan, multi-body simulations, condition indicator

## Abstract

This paper presents methods for continuous condition monitoring of railway switches and crossings (S&C, turnout) via sleeper-mounted accelerometers at the crossing transition. The methods are developed from concurrently measured sleeper accelerations and scanned crossing geometries from six in situ crossing panels. These measurements combined with a multi-body simulation (MBS) model with a structural track model and implemented scanned crossing geometries are used to derive the link between the crossing geometry condition and the resulting track excitation. From this analysis, a crossing condition indicator $C_{\lambda_{1}-\lambda_{2},\;\gamma }$ is proposed. The indicator is defined as the root mean square (RMS) of a track response signal *γ* that has been band-passed between frequencies corresponding to track deformation wavelength bounds of $\lambda_{1}$ and $\lambda_{2}$ for the vehicle passing speed (*f* = *v*/ λ). In this way, the indicator ignores the quasi-static track response with wavelengths predominantly above $\lambda_{1}$ and targets the dynamic track response caused by the kinematic wheel-crossing interaction governed by the crossing geometry. For the studied crossing panels, the indicator $C_{1-0.2\;m,\;\gamma }$ ($\lambda_{1}=1$ and $\lambda_{2}=0.2$) was evaluated for *γ* = *u*, *v*, or *a* as in displacements, velocities, and accelerations, respectively. It is shown that this condition indicator has a strong correlation with vertical wheel–rail contact forces that is sustained for various track conditions. Further, model calibrations were performed to measured sleeper displacements for the six investigated crossing panels. The calibrated models show (1) a good agreement between measured and simulated sleeper displacements for the lower frequency quasi-static track response and (2) improved agreement for the dynamic track response at higher frequencies. The calibration also improved the agreement between measurements and simulation for the crossing condition indicator demonstrating the value of model calibration for condition monitoring purposes.

## 1. Introduction

In a railway network, the components that enable trains to switch from one track to another are called switches and crossings (S&C) or turnouts. This functionality comes at a cost as S&C feature load-inducing rail discontinuities that cause much higher degradation rates compared to regular plain line track. The high degradation rates of S&C is the reason why railway infrastructure managers spend from tens to hundreds of millions of Euros annually on their maintenance. It has been estimated that there are over 300,000 S&C in the EU27 countries and that the maintenance cost per S&C is equivalent to that of approximately 0.3 km of the plain line track [1]. The annual maintenance cost for around 12,000 S&C in Sweden is estimated to be 400–450 MSEK (~40–45 MEUR) [2]. In the United Kingdom, the corresponding cost in 2012 for the approximately 20,000 S&C was 189 MGBP (~212 MEUR), with an additional cost for renewals of 220 MGBP (~246 MEUR) [3]. The number of S&C in track and the costs involved create a potential business case for remote condition monitoring systems, if they can improve maintenance decisions compared to what can be achieved from periodic inspection intervals with measurement vehicles or visual inspection by track engineers. To this end, this paper addresses the development of a model-based condition monitoring system for the monitoring of crossing running surface geometry and ballast properties. It is also worth noting that the degradation of S&C can have a negative influence on operational safety [4], and that remote condition monitoring can constitute an improvement also in this regard as faults can be detected earlier.

**Figure 1:**
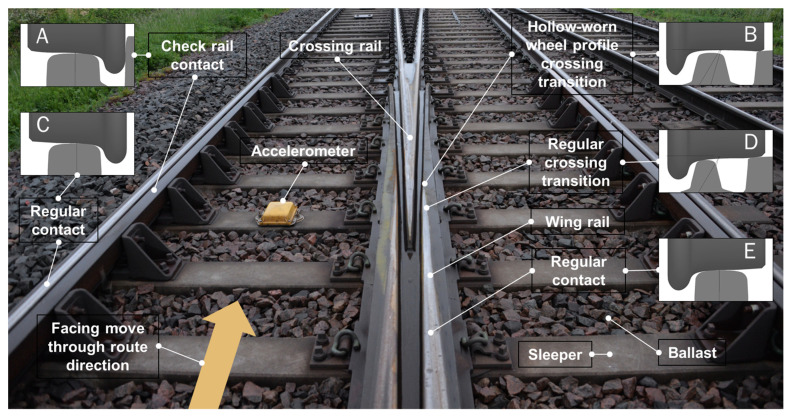
Illustration of a wheelset transition through the crossing panel with the nomenclature for the main components and wheel–rail contact states.

Figure 1 presents a crossing panel with the main components labelled and examples of contact conditions for different wheel profiles in transition. The examples of wheel–rail contacts concern a vehicle passing through the crossing panel in the facing direction in the through (straight) route as indicated by the arrow at the bottom of the figure (trains coming from the opposite direction travel in the trailing move). The branching track to the right is the diverging route. Regular wheel–rail contact conditions experienced by wheels on each rail side before the transition are illustrated with cross-section details C and E. As the vehicle moves further towards the crossing, for the wheel on the right, the transition from wing rail to crossing nose (detail D) will be associated with an impact load on the crossing nose. This is due to a change of direction in the vertical wheel displacement trajectory from a slightly downward motion while rolling on the wing rail to a slightly upward motion on the crossing nose. If the wheel is hollow-worn (concave tread), the given transition occurs later (detail B) and it usually causes much higher dynamic impacts compared to a regular transition. The main contact for the wheel on the left side is the regular contact with the rail, but the back of the wheel flange might come into contact with the check rail (detail A). The check rail is an important guiding mechanism to ensure that the wheelset cannot move too far to the right and make improper interference contact between the right wheel and the tip of the crossing nose.

As a consequence of the impact force on the crossing nose, an accumulation of track damage and degradation follows from each wheel passage through the crossing panel. Wear, plastic deformation, and rolling contact fatigue change the running surface of the crossing nose and wing rails. Ballast deterioration and settlement affect sleeper support conditions and track geometry. Such accumulations of damage will lead to changes in the dynamic response of the structure that can be monitored via sensors in the track [5] and can be used for condition assessment.

The instrumentation concept used for the present study is an accelerometer mounted on the sleeper next to the crossing transition, as shown in Figure 1. The sensors provided by Konux GmbH are installed in the Swedish railway network as a part of Trafikverket’s (the Swedish Transport Administration’s) research activities on remote condition monitoring of crossing panels initiated in the EU-funded In2Rail project [6]. The presented investigations used data from these sensors.

Previous studies have presented a clear correlation between crossing geometry degradation and increased rail and sleeper accelerations. For example, Ref. [5] reported that accelerations measured in a damaged crossing were on average 70% higher compared to a crossing geometry where the running surface had recently been repaired. Previous studies based on simulations have also presented a clear correlation between crossing geometry, wheel–rail contact force, and track response [7,8]. At least three studies [9,10,11], have contrasted measured accelerations at the crossing transition to simulation results. Additional studies on the simulation of dynamic vehicle–track interaction at the crossing transition can be found in [12,13,14].

Further, several studies used only experimental data processing to characterize the magnitude and frequency contents of the crossing rail accelerations. In [5,10] peak values were used, in [9] spectrograms were used, and in [15] wavelet transform was used. Ref. [16] proposed a separation of the measured track response into quasi-static and dynamic domains based on deformation wavelength regions to monitor the ballast condition and crossing geometry, respectively.

It is, therefore, established that a change in crossing geometry condition will lead to a change in track response and that this can be used for condition monitoring purposes. The question is, rather, how this can best be accomplished and what precision can be achieved in identifying the state of the crossing geometry independent of other influencing track and traffic parameters. For this purpose, the present paper used accelerometer data from six different crossing panels combined with concurrently measured crossing geometries and multi-body simulation (MBS) models to establish a more precise link between measured track response and crossing geometry condition. Firstly, the dynamic vehicle–track interaction is simulated using measured crossing geometries to establish the links between the status of crossing geometry and the magnitudes of wheel–rail contact forces and track response. The simulated vertical wheel–rail contact force is used as an indicator of the crossing geometry condition. The scatter in operational conditions is accounted for by simulating dynamic vehicle–track interaction for a range of differently worn wheel profiles. Secondly, the agreement between measured and simulated track responses is evaluated. By comparing these responses, the possibility of monitoring crossing geometry status via embedded sensors and condition indicators is addressed. Thirdly, the difference in agreement between simulation results when using nominal track parameters or track parameters that are calibrated to the different sites is determined. Building on the findings in [16], the track response is separated into quasi-static and dynamic domains based on deformation wavelength regions. Finally, a crossing geometry condition indicator is proposed and evaluated based on the track response in a specified wavelength interval in the dynamic domain.

Additionally, it is important to emphasize that the influence of unknown operational variabilities needed to be excluded to enable interpretations of the crossing condition through its dynamical response. The variations stem from unknown axle load, train speed, and wheel profiles as well as environmental effects on the track superstructure, ballast, and sensor. Because of this, only acceleration measurements stemming from passages with the cabin car bogie in X2 passenger trains (with a known axle load) are used to compare responses between crossing panels. With the tools developed in [16], train speed is identified from the measured acceleration responses. The influence of environmental conditions is not accounted for in this paper as the measurements do not concern long-term data. For example, ref. [17] addresses this problem in detail.

For the monitoring of crossing panel faults that are not easily observed with the present sensor, there are other emerging technologies such as image processing that can identify rail and check rail misalignments and missing bolts in rail to sleeper connections. The inspection images could come from cameras mounted onboard trains [18] or drones.

## 2. Measurement Data

This section presents the measurement data used as the basis for the present investigations. It concerns crossing running surface geometry scans and sleeper accelerations from six crossing panels on the southern mainline in Sweden. The measurements were performed in the first week of June 2019 and are concurrent in time to provide a direct comparison between crossing geometry conditions and dynamic track response.

### 2.1. Crossing Rail Geometry Scans

Geometry scans were taken from three locations on the southern mainline in Sweden, named after their adjacent villages: Höör, Stehag, and Vätteryd. Two crossings were scanned at each location. In this paper, the crossings are labelled with their network IDs for Höör [HO 21B, HO 22A], for Stehag [SG 21A, SG 21B], and for Vätteryd [VAD 102, VAD 131]. The designs of the crossing panels differ among the locations. In Höör, the latest generation of turnouts (EV-60E) is installed, featuring rail fastenings with a soft resilient element (rail pad) between crossing rail and sleepers, while Stehag and Vätteryd feature an older design (UIC60) with a direct and very stiff connection between crossing rail and sleepers. More details of the scanned crossing panels are presented in Table 1.

**Figure 2:**
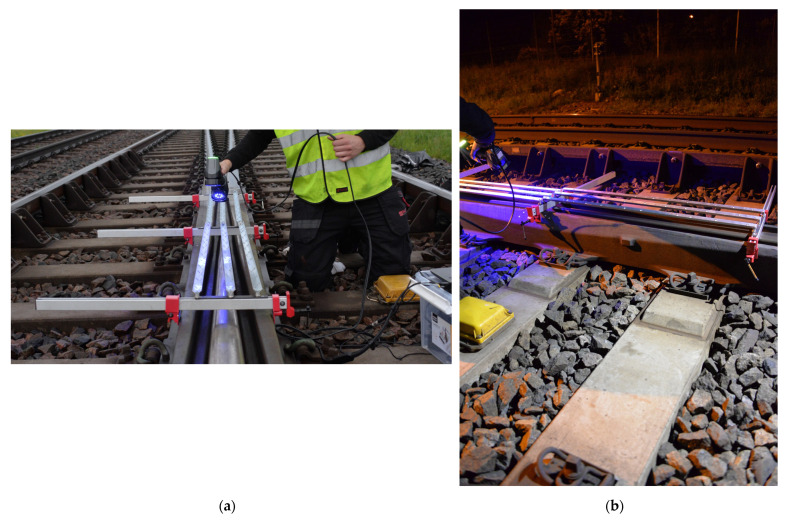
(**a**,**b**) The 3D geometry scan of a crossing transition with Creaform HandySCAN 3D laser scanner [19].

The crossing rail geometry is obtained using the high-precision Creaform HandySCAN 3D laser scanner (see Figure 2b). During the in situ scanning, additional reference objects were mounted on the crossing rail to increase the quality of the scan. The properties of the scanner are given in Table 2.

#### 2.1.1. Processing of Crossing Rail Geometry Scans

**Figure 3:**
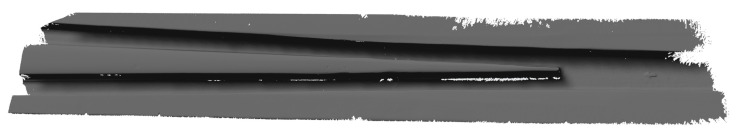
Example of raw 3D geometry scan, crossing panel VAD 131.

In the MBS software, the geometry is provided as a sequence of 2D, cross-section profiles that are orthogonal to the lengthwise direction of the track. The scanned geometry, on the other hand, is a point cloud meshed with polygons to create a surface. One example of raw data from the 3D scan of the VAD 131 crossing is shown in Figure 3. In the figure, holes in the surfaces can be observed and there are also outlying geometry points due to measurement noise.

To prepare the scanned geometry for MBS simulations, the following steps are taken:

- Detection and removal of outlying data points with a fine point distance tolerance for meshing surfaces.
- Orienting the geometry in space to the defined reference frame of the MBS.
- Interpolation of cloud point data to a specified horizontal grid (nearest-neighbor method [20], 0.1-mm horizontal and 1-mm longitudinal spacing).
- Smoothening of the data with two decoupled moving average filters (2-mm lateral and 20-mm longitudinal filter length).
- Sampling the data to 2D profiles with a specified longitudinal spacing (chosen longitudinal spacing: 10 mm).

**Figure 4:**
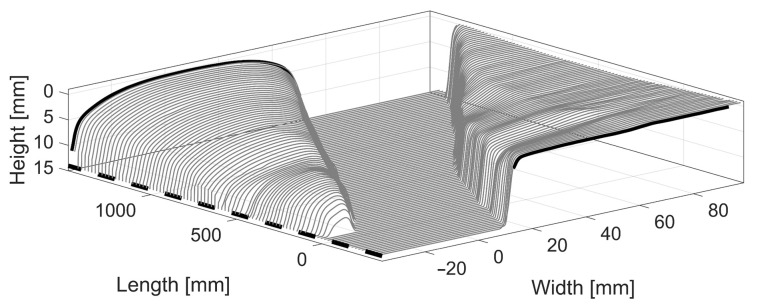
Post-processed, 2D, cross-section profiles ready for MBS analysis, crossing panel VAD 131. The longitudinal spacing between profiles is 10 mm. The crossing nose is to the left in the figure and the wing rail is to the right.

Figure 4 shows post-processed, 2D, cross-section profiles ready for MBS analysis. The 10-mm longitudinal spacing is found to be sufficient as a finer discretization did not alter the simulation results up to 1000 Hz when observed in the frequency domain.

#### 2.1.2. Post-Processed Crossing Rail Geometries

**Figure 5:**
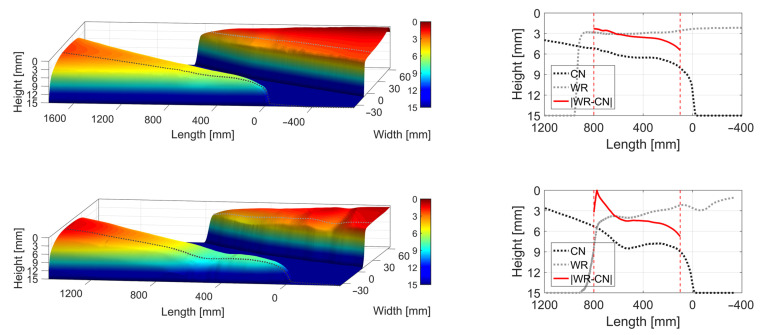
Crossing panel HO 22A (**top**) and VAD 131 (**bottom**): crossing transition 3D scan (**left**) and longitudinal height profiles in transition region for the crossing nose and the wing rail (**right**). The crossing nose (CN) is to the left and the wing rail (WR) is to the right. The longitudinal cross-sections (between vertical, dashed lines) at representative lateral positions illustrate profile degradation.

Two examples of the post-processed, scanned crossing rail geometries are presented in Figure 5. In the figure, the dotted lines that intersect the crossing nose and wing rail surfaces are representative estimations for the lateral wheel–rail contact positions. The subfigures on the right show the extracted height profiles and their absolute difference in the approximated transition region.

The two crossing rail geometries show different states of degradation of the running surface. The HO 22A geometry in the top of Figure 5 shows very few signs of geometry change, while VAD 131 (Figure 5, bottom) shows signs of significant geometry change. The height profile difference between the crossing rail and the wing rail at 500 mm from the crossing nose for HO 22A is around 3.5 mm, while for VAD 131 it is around 4.5 mm. Based on this significant profile difference in conjunction with the observed waviness of the rail surfaces, it is concluded that VAD 131 is in a severely degraded state.

### 2.2. Sleeper Acceleration Measurements

The track response measurement data used for this study are vertical sleeper acceleration time histories recorded during train passages. The accelerations were acquired with a sensor design from Konux GmbH [21]. This sensor is a cellular Internet of Things (IoT) device with long battery life used for the purpose of continuous remote condition monitoring. It is permanently mounted on the sleeper next to the crossing transition (see Figure 1). The sensor properties are given in Table 3. This study used the acceleration data directly from these sensors as input. This means that the only processing applied to the signals before the processing described in this paper is an anti-aliasing filter. An additional important note is that the longitudinal location of the sensor with respect to the crossing transition varies for the six instrumented crossing panels due to different sleeper dispositions.

The received data are from a monitoring period of 7 days in June 2019. In total, the data contains approximately 600 passages of X2 passenger trains (100 per crossing panel). For crossing panel VAD 131, a majority of the train passages (~70%) were discarded for displacement reconstruction due to accelerations exceeding the measurement range of ±100 g. A saturated signal makes displacement reconstruction impossible as the measured response is no longer physical. It can only be used to conclude that the acceleration levels surpassed ±100 g.

**Figure 6:**
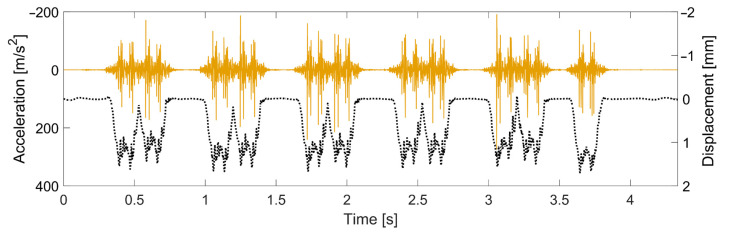
Example of acceleration recording (continuous line) and reconstructed displacement (dotted line).

One example of a recorded acceleration signal and the corresponding reconstructed displacement signal is shown in Figure 6. The displacement reconstruction is performed using the method presented in [16]. It is based on frequency-domain integration, an optimization of a high-pass filter frequency, and a reconstruction of the displacement under each bogie passage with respect to an assumed “at rest position” for the unloaded track between bogie passages.

## 3. Model for Simulation of Dynamic Train–Track Interaction

The dynamic interaction between vehicle and crossing panel is modeled and simulated using multi-body simulations (MBS). The analyses are performed with the commercial software Simpack (v.2019). The track model is a finite element model with all the rails and sleepers modeled by Timoshenko beam elements that are condensed to super elements. Each sleeper is supported by a discretized system of independent bushings in the vertical direction that represents the stiffness and viscous damping of the ballast and subgrade (Winkler bed). Each connection between sleeper and rail is modeled by a single bushing element representing the rail fastening. The degrees of freedom for the nodes of the rails and sleepers are partially constrained to reduce the size of the model. The sleeper nodes can only deform vertically, while the rail nodes can deform vertically and laterally, including their corresponding rotations. The model is generated with an S&C model generation script [6] and implemented in Simpack using its non-linear flextrack functionality. It is found that a track model length of 37 sleepers is sufficient to simulate the dynamic vehicle–track interaction and that a longer track model does not change the results significantly [16].

**Figure 7:**
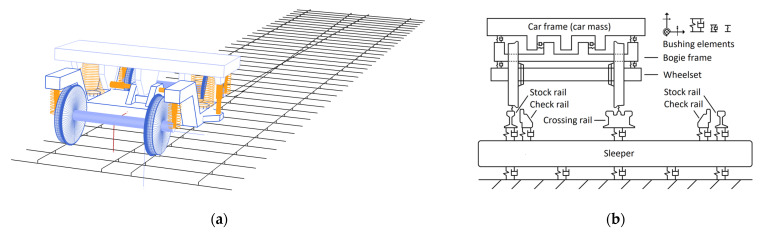
(**a**) MBS model of vehicle–track system, and (**b**) 2D representation of vehicle–track system during a passage through the crossing panel.

The crossing rail geometry is implemented into Simpack by 2D profiles with a 10-mm spacing discretized from the 3D scans of the crossing rail (see Figure 4). The software builds a 3D rail geometry from the 2D sections via a cubic spline interpolation. Additionally, a nominal generic crossing from the S&C benchmark study [22] is used as a reference case. An equivalent linearized Hertz contact is used for the normal wheel–rail contact, while FASTSIM [23] is used to model the tangential contact. The 3D and 2D views of the vehicle–track model are shown in Figure 7. The train speed for the nominal case analysis is 160 km/h, and the simulations start from a static equilibrium position for the vehicle–track system.

### 3.1. Sleeper Void Model

**Figure 8:**
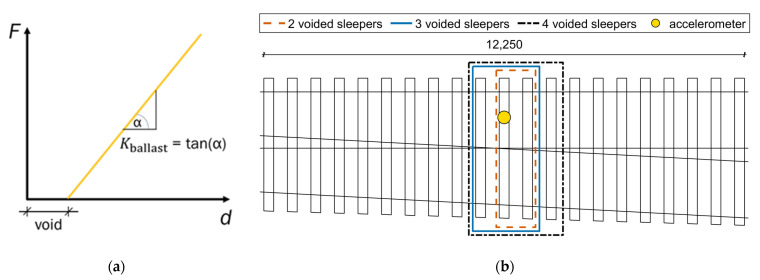
(**a**) Bi-linear force-displacement (*F* versus *d*) function for ballast force per meter sleeper length used for simulation of a voided sleeper. (**b**) Technical drawing for 12.25 meters of crossing panel marking three alternatives for voided sleepers in the MBS model (three cases with 2, 3, or 4 consecutive voided sleepers in the crossing transition).

The decision to include a non-linear ballast support stiffness model accounting for sleeper voids was derived from observations of high magnitudes of the measured sleeper displacements (reconstructed from accelerations) together with the displacement patterns that could only be matched with non-linear ballast stiffness in the simulations. Previous field experience also showed a significant influence from sleeper voids on rail accelerations and displacements [24]. To allow for the MBS model to capture these phenomena, ballast stiffness bushing elements that account for voids between sleeper and ballast are introduced. Figure 8a shows the bi-linear bushing element function implemented for this purpose. Three sleeper void configurations are considered (see Figure 8b). The first case applies a void along the full length of two consecutive sleepers adjacent to the crossing transition. The second case applies the void along the full length of three consecutive crossing sleepers, while the third case applies the void for four consecutive crossing sleepers. This sleeper void model excludes the damping of the bushing element, as the used bushing element damping is defined by its velocity independent from the bushing displacement. Uniform support conditions are assumed along the length of each sleeper. This modeling simplification is observed in the form of addressing averaged track properties (homogenized).

### 3.2. Wheel Profile Data

**Figure 9:**
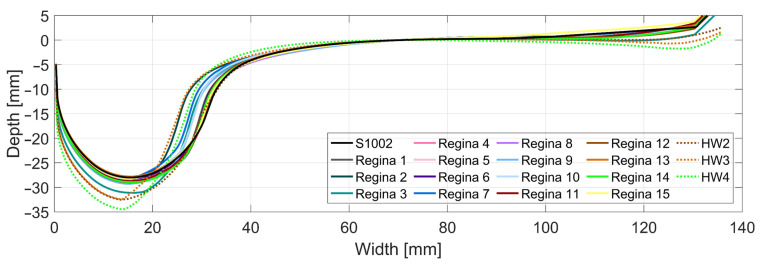
Wheel profile set used in the MBS analysis. Nominal wheel profile S1002 is marked with black color and the scanned Regina passenger train wheel profiles are marked with continuous colored lines, while the three hollow-worn wheel profiles are marked with dashed lines.

A set of wheel profiles is utilized in the simulations to account for the variation in wheel profile shapes present in traffic and to obtain a more representative evaluation of the dynamic vehicle–track interaction at the crossing transition. The set consists of a nominal S1002 profile, 15 measured profiles from Swedish Regina passenger trains [25], and three measured hollow-worn wheel profiles [26]. From these profiles, the nominal S1002 and the Regina profiles are expected to be the most representative of the profiles present on X2 trains. The hollow-worn profiles were included to illustrate their effect on the wheel–crossing interaction but are most commonly found on freight wheels that see less frequent maintenance. The Regina profiles are sampled from a larger set of 279 profiles to save simulation effort. The 15 profiles uniformly cover the range of global cone angles (φ) present in the original sample, where φ is defined as the height difference between the flange root and the field side of the wheel profile normalized by their lateral spacing [27]. Here, the flange root and field-side heights of the profile are represented by the profile height at width coordinates 45 and 125 mm, respectively, as shown in Figure 9. These reference points are used as they correspond to typical lateral contact locations on crossing nose and wing rail for each wheel profile. Thus, these points determine the longitudinal location for transition between wing rail and crossing nose, and there is a strong correlation between the cone angle φ of a given wheel profile and the transition location [27]. By ensuring a large and uniform spread in φ available from the wheel profile sample, the broadest possible range of transition locations is evaluated for the measured crossing geometries.

### 3.3. Simulation Setup

The MBS analysis presented in this paper is based on three simulation cases. The first case uses the nominal vehicle and track model parameters that are presented in Table 4. The second case is based on the first but uses soft rail pads instead of the stiff direct fixing between crossing rail and sleepers. Lastly, the third case uses the calibrated track model parameters that will be presented in Section 6. The simulations cover different crossing rail geometries, wheel profiles, and train speeds. A summary of the studied load cases is presented in Table 5.

## 4. Observation of Track Response from Crossing Impact Loading

When a wheel rolls over a crossing, the track (and vehicle) is excited in a broad frequency range. To formulate a suitable indicator of crossing geometry condition computed from a track response signal, a fundamental understanding of the most relevant frequency ranges for dynamic wheel–crossing interaction is of the essence. On the low end of the frequency spectrum is the quasi-static track displacement stemming from the moving static wheel load. Based on a beam on elastic foundation model for a plain line track, it has been shown that the quasi-static track response in the longitudinal direction is more or less fully described by track deformation wavelengths longer than one meter (see [29]). At the typical speed of 160 km/h for the X2 train considered in this study, a 1-meter wavelength corresponds to a dynamic excitation of 44 Hz. For crossings, it was demonstrated in [16] that the quasi-static track deformation vanished if the sleeper displacement was spatially high-pass-filtered with a cutoff at a wavelength of 1 meter.

**Figure 10:**
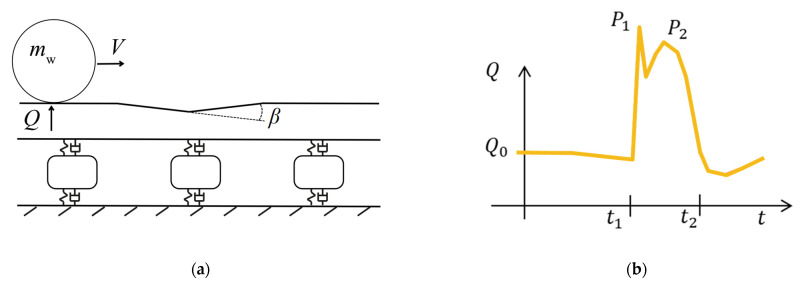
(**a**) Schematic vertical-longitudinal view of the vertical wheel displacement trajectory over a dipped joint or crossing. (**b**) Qualitative illustration of the corresponding time history of the vertical wheel–rail contact force.

Farther up in the frequency spectrum is the dynamic excitation induced by the wheel–rail impact force during the crossing transition. Based on analytical modeling for a dipped joint [30], this force impulse is expected to consist of two main components. The first component is the so-called $P_{1}$ force in the frequency interval 500–1000 Hz, which is due to the wheel and rail oscillating out of phase with a frequency that is related to their contact stiffness. The second component is the so-called $P_{2}$ force, which is related to the wheel and track oscillating in phase on the foundation stiffness (50–100 Hz). Figure 10a,b qualitatively illustrates the wheel trajectory caused by a dipped joint or crossing with dip angle *β* and the resulting time history of the wheel–rail contact force *Q* with the two force peaks. The $P_{1}$ and $P_{2}$ force peaks are proportional to *β*, which describes the directional change required by the wheel while rolling over the dip. This dependence can be found in [30] or can be derived from the principle of linear momentum.

Naturally, the overall dynamic response of the real structure can be expected to be broader with a wider range of excitation frequencies, especially for an irregular structure such as the crossing panel and due to wheel out-of-roundness and irregularities in track geometry. However, for typical system properties, these analytically derived force components indicate the frequency regions of interest and are expected to qualitatively represent fundamental forces and frequencies of the real system. The existence of a local maximum in the contact force due to the $P_{2}$ force is supported by measurement data. Based on the vertical wheel–rail contact force measured with an instrumented wheelset in a crossing [31], the duration of the $P_{2}$ force impulse on the crossing nose could be estimated to be about 8 ms. Measurements in a crossing instrumented with strain gauges [32] suggested a duration of the $P_{2}$ force impulse to be about 10 ms. The time duration of these impulses thus indicates phenomena in the order of 100 Hz. Based on wavelet analysis of measured crossing rail accelerations due to wheel impact, the most dominant spectral energy peaks were found at around 70–80 Hz (see [15]).

Considering the 2-kHz sampling rate of the data used in this study, the measured accelerations encountered limitations to successfully resolve the frequency domain of the *P*_1_ force (500–1000 Hz). As the acceleration signals are short and transient, eight sampling points are considered to be sufficient for sampling one full oscillation of the highest frequency component of the signal. With this recommendation, the low-pass cutoff frequency is set to 250 Hz (for the 2-kHz sampling rate).

**Figure 11:**
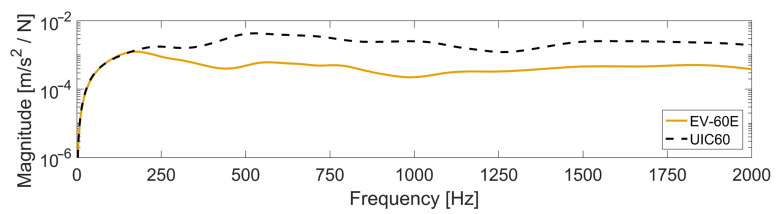
Crossing sleeper accelerance FRF (at sensor position) for the load applied on the crossing rail above the sleeper. Results are shown for two turnout designs, EV-60E, featuring rail fastenings with a soft resilient element between crossing rail and sleepers, and UIC60 with a direct and very stiff connection between crossing rail and sleepers. Nominal ballast parameters were used (see Table 4).

In addition to the sampling frequency, the location of the sensor itself must be accounted for. Figure 11 presents the simulated accelerance (acceleration over force) frequency response function (FRF) at the sensor location on the sleeper for a load applied on the rail at the crossing transition. It can be observed that there is a separation in response between the two studied S&C designs at around 150 Hz. This difference in response is explained by the difference in coupling stiffness between crossing rail and sleepers. The lower rail pad stiffness of the EV-60E results in a dynamic decoupling of crossing rail and sleeper at a lower frequency and lower acceleration levels at the sensor. For condition monitoring purposes, this needs to be accounted for, as the same crossing geometry would lead to different track responses and, therefore, sensor readings depending on the S&C design. It can further be noted that the frequency dependence of the accelerance created a weighted response at the sensor, which makes it more difficult to create a condition indicator exactly proportional to the contact force at the crossing. No efforts were made to account for this effect in the present study.

To summarize, the frequency range of interest for crossing geometry monitoring starts at frequencies corresponding to a deformation wavelength of 1 m (44 Hz at 160 km/h). At the upper end, the sampling frequency limits the observable frequency range and the upper cutoff is, therefore, chosen as deformation wavelengths of 0.2 m (220 Hz at 160 km/h). This wavelength range will, thus, box in the frequency range around the $P_{2}$ force while short transients related to the $P_{1}$ force or local irregularities cannot be observed. This is a limitation, but the considered frequency range is the most relevant for damaging track deformation stemming from the crossing impact as practically all of the sleeper displacement energy is described by frequency content up to a wavelength of 0.2 m [16]. To observe the dynamics in the relevant frequency range and for the full length of the crossing, a condition indicator $C_{\lambda_{1}-\lambda_{2},\;\gamma }$ is defined as the RMS of the band-passed signal for a bogie transition where the band-pass frequencies correspond to deformation wavelength bounds of $\lambda_{1}$ and $\lambda_{2}$. For the present investigations, the bounds are chosen as $\lambda_{1}$ = 1 m and $\lambda_{2}$ = 0.2 m, as outlined above to suit the current line speed and sampling frequency. The indicator $C_{1-0.2\;m,\;\gamma }$ is evaluated for *γ* = *u*, *v*, or *a* as in displacements, velocities, and accelerations, respectively. If the condition indicator were to be applied to other line speeds, the $\lambda_{1}$ limit would be expected to still be valid as it is motivated by observations in the wavelength domain while $\lambda_{2}$ should be adjusted to cover the frequency range of interest.

## 5. MBS Analysis with Nominal Track Model Parameters: Influence of Crossing Geometry

In this section, MBS results are presented for simulations with nominal track model parameters and seven crossing geometries (six measured, one nominal). Results are for the UIC60 design (simulation case 1, the stiff connection between crossing rail and sleepers (see Table 4)) unless otherwise mentioned. Comparisons to the EV-60 S&C design (simulation case 2, the soft connection between crossing rail and sleepers) are made when required. The purpose of these simulations is to present an overview of the variability in the dynamic wheel–crossing interaction for different crossing geometries, wheel profiles, rail to sleeper connection stiffnesses, and to demonstrate the crossing geometry condition indicators before model calibration. Comparisons between simulations and measurements are presented in Section 6 and Section 7.

**Figure 12:**
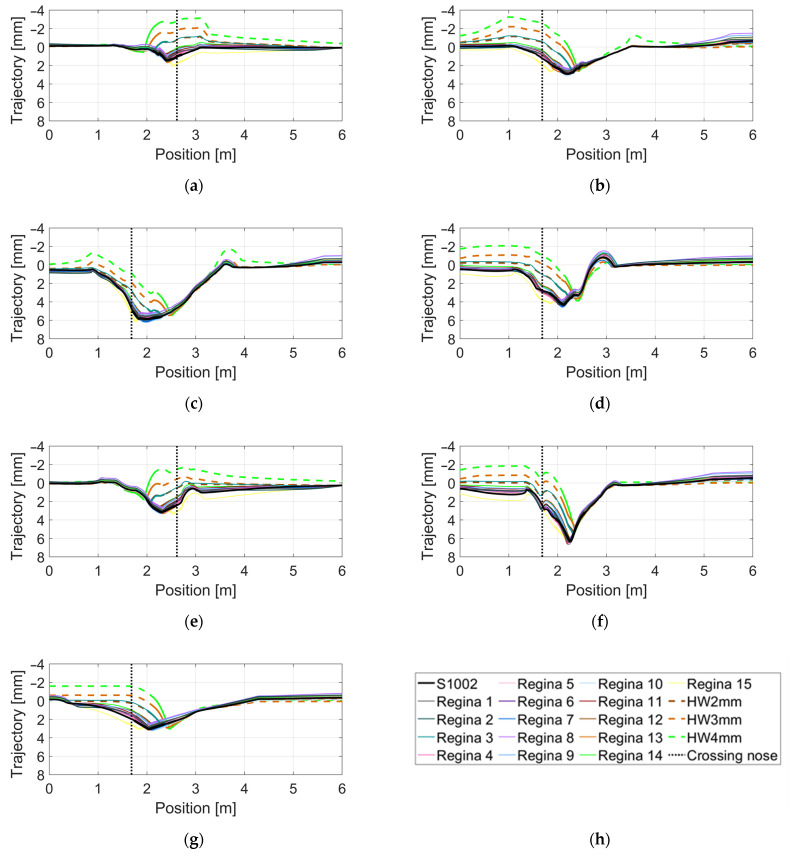
Calculated trajectories of relative vertical wheel–rail displacement for 19 different wheel profiles in seven different crossings: (**a**) HO 21B, (**b**) HO 22A, (**c**) SG 21A, (**d**) SG 21B, (**e**) VAD 102, (**f**) VAD 131, (**g**) Nominal crossing geometry, (**h**) wheel profile legend (vertical, dotted line is the tip of crossing nose).

In Figure 12, the relative vertical wheel–rail displacement is presented for each crossing panel. All subfigures contain trajectories for the 19 different wheel profiles. Figure 12a,e are for the case of transition in the trailing move, while the other subfigures are for transition in the facing move. The trajectories for the three hollow-worn wheel profiles are plotted with dashed lines. It is observed that the largest change in trajectory direction occurred for hollow-worn wheels that tended to have a later transition from wing rail to crossing nose. In Figure 12f for the VAD 131 crossing panel, the deepest wheel trajectory is observed. This confirms that VAD 131 has the highest level of degradation among the analyzed crossing panels. Further, the dip angles are computed for each combination of wheel profile and crossing. The dip angle is defined as the difference in inclination between two linear regression lines fitted to the wheel trajectory in the ±15-cm region from the point of the transition (see Figure 10a). It should be noted that these dip angles are local approximations and that the studied in situ crossings do not have the idealized wheel trajectory dip shown in Figure 10. For all practical purposes, the dip angle approximations and other aspects of the wheel–crossing interaction kinematics are the same regardless of the stiffness in the crossing to sleeper connection.

**Figure 13:**
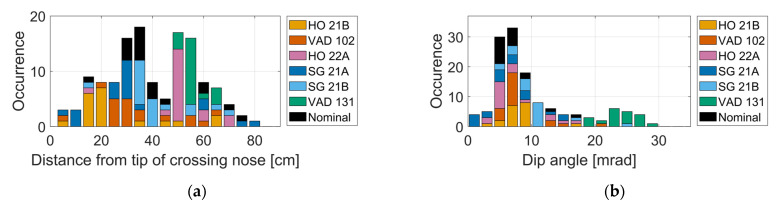
Crossing transition geometry indicators: (**a**) longitudinal position of wheel transition relative to the tip of the crossing nose, and (**b**) dip angle for the reversal in vertical wheel trajectory during the transition. The results correspond to 19 wheelsets passing through seven crossing panels.

In Figure 13, stacked histograms are presented for two kinematic indicators of crossing geometry condition. Figure 13a shows the simulated longitudinal position of the wheel transition relative to the tip of the crossing nose. For crossing panel VAD 131, all transitions are gathered within a small region. In [5], a so-called fatigue area criterion was established to characterize the state of the crossing geometry. The fatigue area is defined as the crossing nose region where most of the transitions occur. For a short fatigue area in the lengthwise direction of the track, the crossing transition impacts would be condensed, indicating a worn crossing geometry with larger dip angles.

In Figure 13b, the stacked histograms show the computed dip angles. It is observed that VAD 131, which is in a severely worn state, experiences the largest dip angles. According to the theory presented in Section 4, the wheel–rail impact force is proportional to the dip angle. Thus, crossing panels that encounter large dip angles will be subjected to higher wheel–rail contact forces and, as a consequence, higher degradation rates. Overall, for all seven crossing geometries and 19 wheel profiles, substantial variability in crossing transition locations and dip angles is observed. Thus, simulating operational conditions that account for the true geometry of wheel and rail is necessary to quantify the state of the crossing geometry. From these two subfigures, it can be expected that the VAD 131 crossing panel is subjected to a higher degradation rate and is in a worse condition than the other crossing panels. The other measured crossings indicated a behavior that is similar to the crossing panel with a nominal crossing geometry.

**Figure 14:**
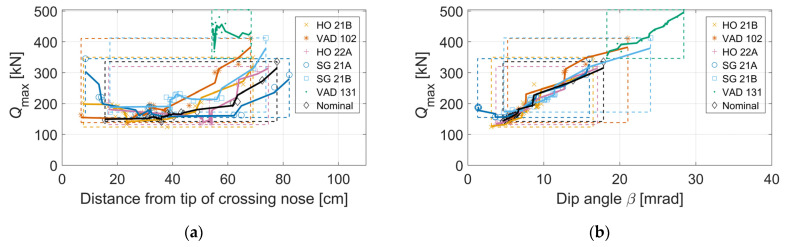
Correlation between *Q*_max_ on the crossing rail for the leading wheel and (**a**) longitudinal position of wheel transition relative to the tip of the crossing nose, (**b**) dip angle. Simulations are computed for seven crossing panels, 19 different wheel profiles, and a train speed of 160 km/h. Each scatter point presents the result for a particular wheel profile, while the marker makes a relation to the corresponding crossing panel. Continuous lines present results after the application of a three-point moving average filter for the cloud of the scatter points. Dashed boxes (with the corresponding color) cover the min-max domain of results for each crossing panel. The *Q*_max_ values are taken from results that are low-pass-filtered at 250 Hz.

Other potential indicators of the crossing geometry condition are the correlation between the maximum wheel–rail contact force (*Q*_max_) and the position of the transition (see Figure 14a). The results show that higher contact forces occur for the early and late transitions. For all seven crossing geometries, a small variability in *Q*_max_ is observed when the transition occurs in the region of 15 to 50 cm from the tip of the crossing nose. The high forces for the early transition are related to poor contact conditions close to the tip of the crossing nose. The late transitions, more than 60 cm after the crossing nose tip, are associated with hollow-worn wheel profiles. Overall, the crossing panel VAD 131 encounters later transitions compared to the other crossing panels. This is due to the worn state that causes a larger height profile difference between the wing rail and the crossing nose (see Figure 11). Thus, the tread inclination (cone angle *φ*) of the wheels is not able to gap that height profile difference, and the transition occurs later.

Figure 14b shows the correlation between *Q*_max_ and dip angle. The observed correlation for all seven crossing geometries is mostly linear. This linear relation is strongest for the nominal crossing geometry with smooth wheel trajectories where the dip angle approximation is most accurate. The highest wheel–rail contact forces and dip angles are observed for crossing panel VAD 131, where the span of dip angles is from 17.5 to 28 mrad, while the range of *Q*_max_ is from 350 to 500 kN. The behavior of the other five scanned crossing geometries is similar to the behavior of the nominal crossing rail geometry. The dip angle range is from 5 to 17 mrad, with *Q*_max_ spanning from 120 to 320 kN.

Overall, a large variation in *Q*_max_ is observed for the 19 different wheel profiles. This shows that neither the location of the impact transition nor the dip angle is uniquely defined for a particular crossing geometry. It has previously been shown that the structural loading and the following degradation rate of a particular crossing is strongly related to the *Q*_max_ during the transition [2,8]. Together, this emphasizes the need for simulations of true operational conditions to determine the state of a crossing panel. In addition, it is observed that there is no clear separation in results when simulating facing (HO 22A, SG 21A, SG 21B, VAD 131, Nominal) or trailing moves (HO 21B, VAD 102). The results in Figure 14a,b would be qualitatively the same if they were based on results from simulation case 2 with soft pads, but with lower force magnitudes.

**Figure 15:**
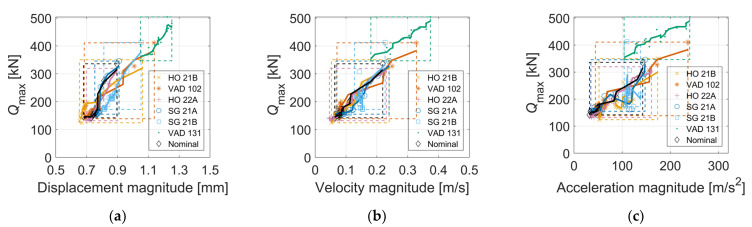
Correlation between *Q*_max_ and maximum (**a**) sleeper displacement, (**b**) sleeper velocity, (**c**) sleeper acceleration. Results are from simulations with 19 different wheel profiles, seven crossing panels, and a train speed of 160 km/h. Wheel–rail contact forces and accelerations are low-pass-filtered at 250 Hz. For interpretation of the details, see the caption of Figure 14.

Figure 15 shows the relations between the maximum vertical wheel–rail contact force and maximum vertical sleeper (a) displacement, (b) velocity, and (c) acceleration. The results show a moderate level of linear dependence. Several previous studies observed that the dynamic track response is highly proportional to the crossing geometry and its dip angle [8,33]. The domain of results for each crossing shows how the crossing geometry influences quantities that are measurable by the embedded accelerometer. Displacement magnitudes span from 0.65 to 1.25 mm, while maximum accelerations span from 30 to 240 m/s^2^. The outlier in the results, with the highest magnitudes, is the crossing panel VAD 131. Moderate magnitudes are observed for crossing panel VAD 102, while similar magnitudes are observed for the other scanned crossings and the nominal crossing geometry.

**Figure 16:**
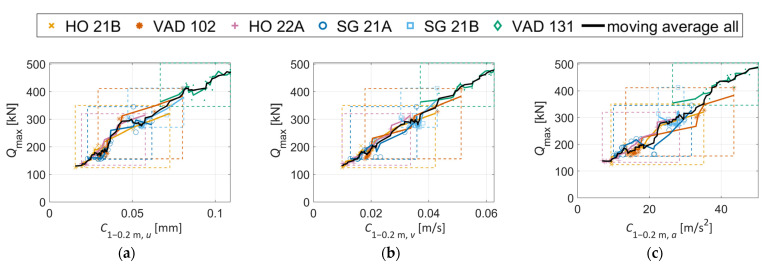

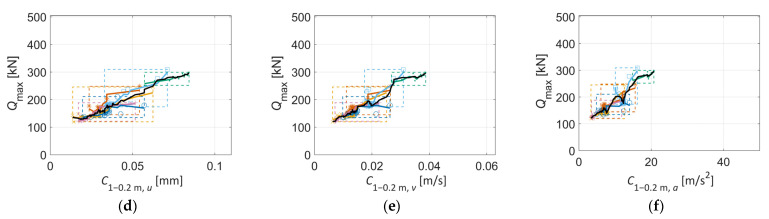
Relation between simulated *Q*_max_ and $C_{1-0.2\;m,\;\gamma }$ for six different crossing panels (nominal parameters, stiff (**a**–**c**) and soft (**d**–**f**) rail to sleeper connection). The black, continuous line presents a 7-points moving average filter through the cloud of all scatter points. Dashed boxes (with the corresponding color) cover the min-max domain of results for each crossing panel.

Figure 16 presents relations between *Q*_max_ and the crossing condition indicator $C_{1-0.2\;m,\gamma }$ (see Section 4) computed for displacements, velocities, and accelerations, for stiff (a,b,c) and soft rail to sleepers’ connection (d,e,f). It is observed that the range of both *Q*_max_ and $C_{1-0.2\;m,\;\gamma }$ is significantly reduced for the latter due to the softer track, but the correlation is strong for both turnout types. It is further observed that these tailored condition indicators display a stronger correlation to *Q*_max_ compared to the relations observed in Figure 15. making them more suitable for the task of crossing condition monitoring. The Pearson correlation coefficients [34] and linear regression mean errors [35] corresponding to the plots in Figure 15 are (a) 0.96 and 10.9%, (b) 0.97 and 8.2%, and (c) 0.88 and 15.6%. For Figure 16 they are (a) 0.97 and 7.0%, (b) 0.98 and 6.0%, (c) 0.97 and 8.7%, (d) 0.96 and 6.5%, (e) 0.96 and 6.0%, and (f) 0.94 and 7.3%).

The investigations will further proceed to observe how the correlations in Figure 16 compare to measurements and how they change if the track models are calibrated to the measured response for each crossing.

## 6. Calibration of MBS Track Model Parameters

This section addresses the calibration of an MBS track model for each of the six crossing panels using the scanned crossing rail geometries and the reconstructed displacements from the concurrently measured sleeper accelerations. The objectives of the calibration are to investigate (1) how well the MBS model can replicate the measured response and (2) whether an improved accuracy for the monitoring of crossing geometry can be achieved.

Sleeper displacement is chosen as the dynamic response for the calibration of track model parameters. This is because it is assumed that both the MBS model and the measurements are the most accurate in the lower frequency region of the track response that contributes most to the displacements. In [16], it was found that displacement wavelengths down to 0.5 meters (89 Hz at 160 km/h) account for 90–99% of the total displacement energy depending on the crossing panel. In addition, the track response was only measured at one location, which gives limited observability of the track deformation. While one measurement location should be sufficient to get an indication of the global track and sleeper deformation patterns, the observability of the system decays at higher frequencies due to local deformation modes. The corresponding levels of correlation between measured and simulated sleeper velocities and accelerations are investigated in addition to the agreement in displacements.

The objective function minimized in the calibration is the RMS of the difference between the measured and simulated time-domain sleeper displacement for an X2 train [36] cabin car bogie passage. Each displacement signature accounts for 10 meters of travelled bogie distance. A single bogie passage using nominal S1002 wheel profiles is used to evaluate the objective in simulations due to the computational cost of the MBS simulations. Each simulation takes 35 to 80 min depending on train speed and model stiffness, using one node of an office desktop computer (i7-8700 CPU @ 3.20 GHz).

The calibration is performed using a brute force grid search optimization performed in two steps. Coarse parameter space is evaluated in the first search to determine a global minimum region and visualize the parameter influence on the sleeper displacement signature. This is followed by a narrower grid search with a finer discretization of the parameters.

In total, seven parameters are used in the calibration. For the bilinear ballast stiffness model (see Section 3), the calibrated parameters included the (1) number of voided sleepers, (2) depth of the sleeper void, (3) linear ballast stiffness when the void is closed, and (4) ballast damping. Additionally, for the EV-60E crossing panels that are designed with a resilient rail pad, the rail pad stiffness and damping are calibrated. Lastly, for the case with very large, measured displacements (VAD 131), a simplification of rail damage is introduced by reducing the bending stiffness of the crossing rail.

Because the number of voided sleepers (0–4) is a parameter that takes an integer value, the problem is non-smooth. Considering this, brute force optimization is considered to be a natural first step in solving the crossing panel MBS model calibration problem. This approach was deemed sufficient for the purposes of this paper where the objective is to investigate the potential in calibration.

The use of a uniform ballast stiffness along the crossing panel length but for the individually adjusted sleeper voids is a simplification. This neglects the fact that ballast properties may vary considerably along the rail track length [29] and laterally along each sleeper. Given that the calibration is based on only one measurement point, however, it is not reasonable to assume that the influence from such variations can be identified from this information. Additionally, as the dominant line speed differed between the crossing panels, the calibrations are performed for different train speeds.

As a final note on the calibration methodology, a time-domain calibration is chosen for two main reasons: (1) Nonlinear models are used for the voided sleeper cases. Therefore, the linear dynamics FRFs are not representative for those models. (b) The model’s FRFs do not account for rail surface geometry information that affects the measured dynamic vehicle–track interaction. Additionally, it is observed from acceleration FRF, given in Figure 11, that the system is highly damped with attenuated resonances. This makes it infeasible to use a resonance-weighted model calibration and creates a more levelled frequency-domain based optimization space for which parameter identification is more difficult. However, a frequency-domain calibration could be performed by transforming the time-domain signals to the frequency domain using a Fourier transform and comparing the agreement for these signals. This would not add any information in comparison to the time-domain based formulated objective function. It would, on the other hand, enable the frequency-dependent weighting of the objective function, which might have some benefits for the optimization control; this alternative is not explored in this study.

**Figure 17:**
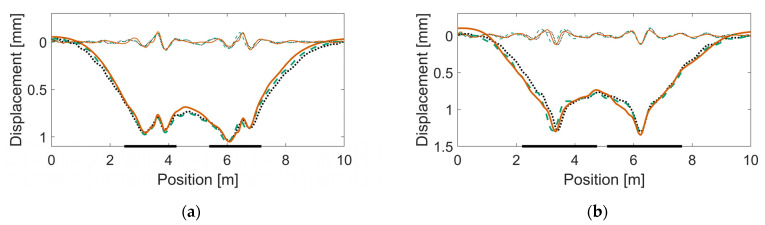
Calibration results showing measured and simulated sleeper displacements (full signals, thick line; and band-passed to 1.0–0.2-m wavelengths, thin line) for an individual bogie passage in crossing panels (**a**) HO 21B (trailing move: soft linear ballast properties) and (**b**) HO 22A (facing move: two voided sleepers). Dotted and dashed lines (black and green) are for measured sleeper displacements, while the continuous line (orange) is for simulated sleeper displacement. The two thick, horizontal lines on the frame of the figures show the regions of the crossing rail scan.

**Figure 18:**
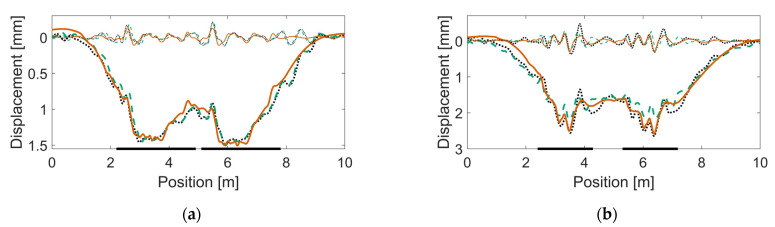
Calibration results showing measured and simulated sleeper displacements for an individual bogie passage in crossing panels (**a**) SG 21A (facing move: three voided sleepers) and (**b**) SG 21B (facing move: three voided sleepers). See also the caption of Figure 17.

**Figure 19:**
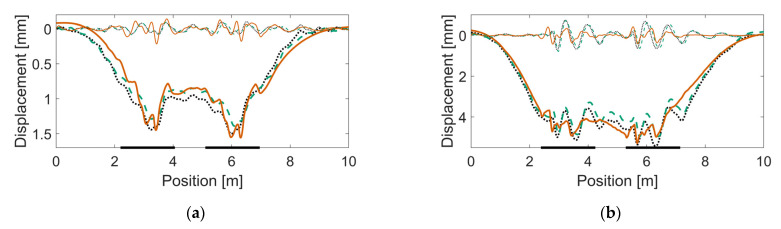
Calibration results showing measured and simulated sleeper displacements for an individual bogie passage in crossing panels (**a**) VAD 102 (trailing move: soft linear ballast properties) and (**b**) VAD 131 (facing move: four voided sleepers and damaged rail). See also the caption of Figure 17.

**Figure 20:**
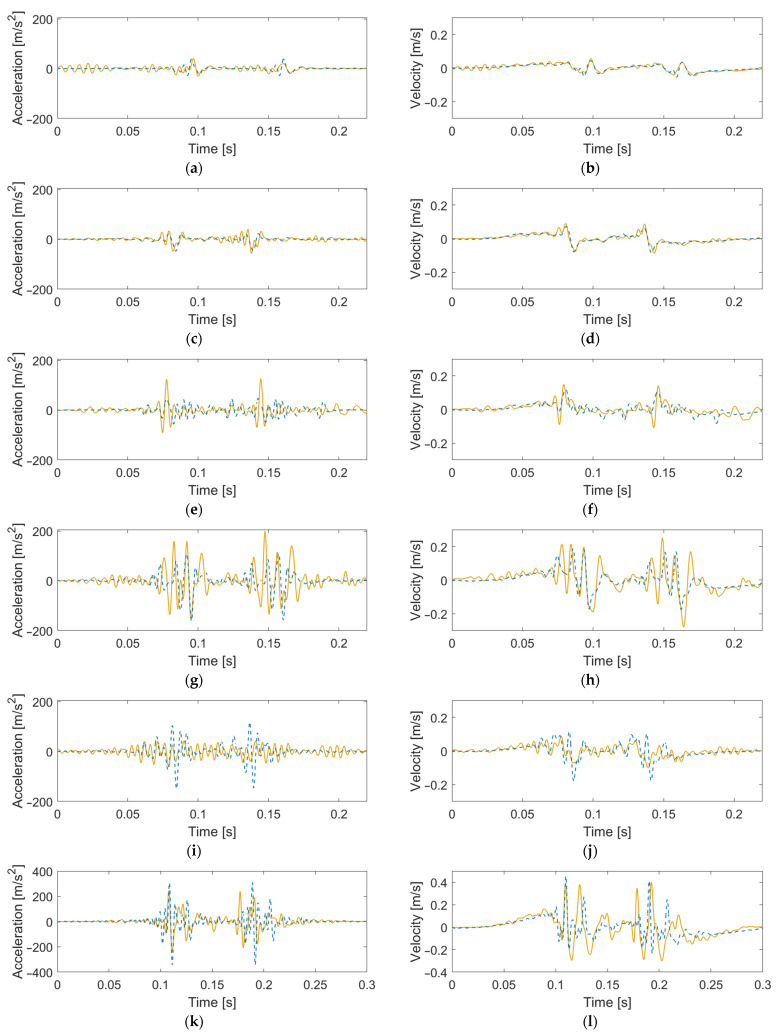
Examples of simulated (dashed line) and measured (continuous line) sleeper acceleration and velocity responses: (**a**,**b**) HO 21B, (**c**,**d**) HO 22A, (**e**,**f**) SG 21A, (**g**,**h**) SG 21B, (**i**,**j**) VAD 102, (**k**,**l**) VAD 131. Note different scale for VAD 131. Train speeds are 160 km/h for HO 21B, SG 21A, and SG 21B; 190 km/h for HO 22A, VAD 102; and 130 km/h for VAD 131.

The resulting track parameter values after calibration are listed in Table 6. In Figure 17, Figure 18 and Figure 19, examples of sleeper displacement signatures are presented. Each figure shows two examples of measured sleeper displacements and one simulated sleeper displacement. Additionally, each figure shows the corresponding displacement signatures that are spatially band-pass filtered to a region of 1.0–0.2-m wavelengths. See [16] for more details of this decomposition. In Figure 20, examples of measured and simulated sleeper velocities and accelerations are presented for the six calibrated models. The presented sleeper velocity and acceleration signals are low-pass-filtered at 250 Hz.

From Figure 17, Figure 18 and Figure 19, it is observed that strong correlation is achieved between the measured and simulated sleeper displacements. In Figure 20, the examples of measured and simulated sleeper accelerations show varying levels of agreement. For the sleeper velocities, the observed agreement is from moderate to high.

**Figure 21:**
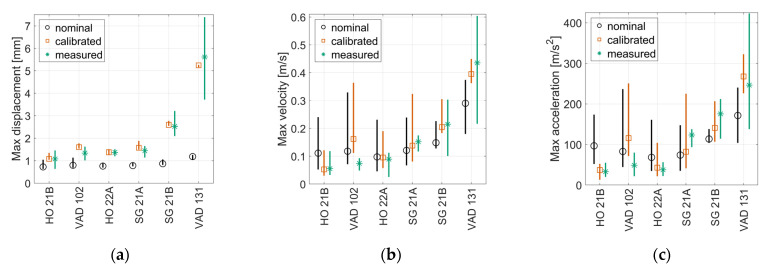
Comparison between maximum measured and simulated sleeper responses (mean value marker, full range vertical line) for (**a**) displacement, (**b**) velocity, and (**c**) acceleration. Simulated results with either nominal (o-black) or calibrated track model parameters (□-orange); train speed 160 km/h for HO 21B, SG 21A, and SG 21B; 190 km/h for HO 22A, VAD 102; and 130 km/h for VAD 131; and 19 different wheel profiles. Measured results from 20 signals (*-green) recorded at similar train speeds as in the simulations. Data are low-pass-filtered at 250 Hz.

Figure 21 shows a comparison of measured and simulated dynamic sleeper response maxima for all crossings. The simulation results were generated using a sample of 19 different wheel profiles (see Section 3 and Figure 9) using either the calibrated or nominal track model parameters. The measurement results were computed from the 20 vehicle passages closest to the dominant line speed out of the 100 measured signals available for each crossing. By selecting a subset of the available measurement signals, all vehicle passages have a speed within approximately ±5% of the dominant line speed, making them more comparable to the simulation results.

The best agreement between measured and simulated responses is observed for the sleeper displacements. This is expected since displacements were used in the calibration. The results from the simulations with nominal track parameters show qualitative trends that are similar to the measured responses. As expected, for most crossings, the simulations with the calibrated track parameters show better agreement with measured responses compared to the simulations with nominal parameters. The only exception is VAD 102, which has a low agreement to measured sleeper velocities and accelerations for the calibrated case and better agreement for the nominal case.

In conclusion, the applied MBS model can achieve a good level of calibration based on the physically scanned crossing rail geometries and the corresponding measured (reconstructed) sleeper displacements. Lower quality of agreement is achieved for the sleeper velocities and accelerations. In Figure 20 it can be observed, especially for the acceleration signals, that there is a higher level of dynamic excitation in the measurements compared to the simulation model. Significant acceleration levels are observed already from the start of the measurement signal, but not in the simulated response. In this case, the leading axle is approximately 3.5 m from the accelerometer, but this region was not covered by the geometry scan. Additionally, when the leading axle is passing through the scanned region, the trailing axle is outside this region (where the MBS model assumes a nominal smooth rail). It is, therefore, concluded that wheel–rail interaction outside the scanned region contributes to the dynamic response of the instrumented sleeper. The current scan lengths range from 1.8 to 2.7 meters. Thus, to calibrate the model based on sleeper acceleration instead of sleeper displacement, the full 10-m crossing panel region should have been scanned, possibly also on the opposite (check rail) side.

There are also additional uncertainties that are not captured by the crossing geometry scans such as long-wavelength track irregularities (irregularities in longitudinal level, lateral alignment, track gauge, etc.) and out-of-round wheels that will influence the wheel trajectory and dynamic track response.

## 7. Agreement between Measured and Simulated Crossing Condition Indicators

**Figure 22:**
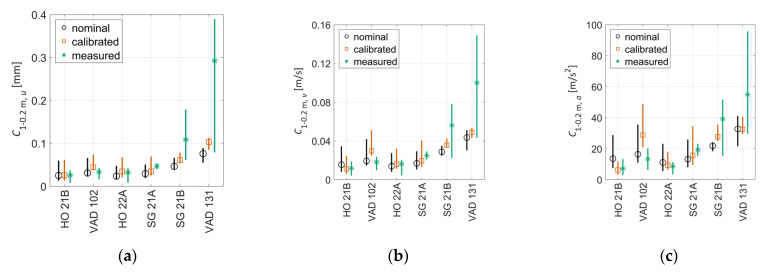
Comparison of $C_{1-0.2\;m,\gamma }$ for measured and simulated sleeper responses (mean value marker, full range vertical line) for (**a**) displacement, (**b**) velocity, and (**c**) acceleration. Simulated results concern nominal (o-black) and calibrated parameters (□-orange) for analyses with one train speed and 19 different wheel profiles. Measured responses (*-green) consist of 20 signals recorded at speeds similar to simulation train speed. Train speeds are 160 km/h for HO 21B, SG 21A and SG 21B; 190 km/h for HO 22A, VAD 102; and 130 km/h for VAD 131.

This section presents a comparison between crossing condition indicators $C_{1-0.2\;m,\gamma }$ (see Section 4) computed from measured and simulated track responses, and with or without calibrated track parameters in the simulations. Figure 22 presents a comparison of $C_{1-0.2\;m,\;\gamma }$ computed for measured and simulated responses. In total, 20 vehicle passages with speeds closest to the simulated speed were chosen to show the level of variability in the measurements. The simulated responses corresponded to simulations with the 19 different wheel profiles to account for operational variability. Based on the average values, it is not possible to make a definite conclusion as to which version of the $C_{1-0.2\;m,\gamma }$ crossing indicator has the better agreement. It can, however, be concluded that all versions show (1) a very good agreement for HO 21B and HO22A and (2) a reasonable agreement for SG21A and SG21B, while (3) the results are more scattered for VAD102 and VAD 131. Here the calibration resulted in a moderate improvement in agreement between measurements and simulation. It is still encouraging that an improved agreement is obtained also for $C_{1-0.2\;m,\;v}$ and $C_{1-0.2\;m,\;a}$ given that the calibration is performed for displacements where the low-frequency track response dominates and the crossing indicator is computed on a band-passed signal at higher frequencies. With the observed levels of agreement, it is concluded that these MBS models can provide qualitative comparisons in crossing geometry condition between different turnouts via the crossing indicator $C_{1-0.2\;m,\;\gamma }$. It also shows promise to deliver results that can be interpreted quantitatively.

**Figure 23:**
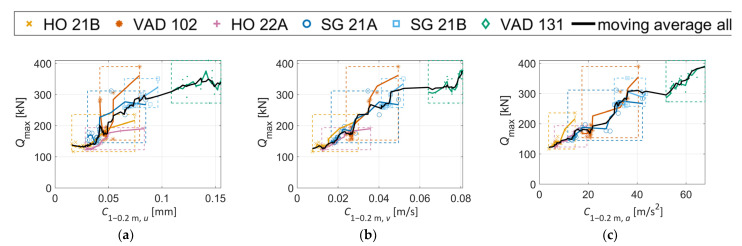
(**a**–**c**) Relation between simulated *Q*_max_ and $C_{1-0.2\;m,\;\gamma }$ for six different crossing panels with calibrated track parameters. The black, continuous line presents a 7-points moving average filter through the cloud of all scatter points. Dashed boxes (with the corresponding color) cover the min-max domain of results for each crossing panel.

Figure 23 shows the correlations between $C_{1-0.2\;m,\;\gamma }$ and *Q*_max_ for calibrated track parameters. In Figure 23a, it is observed that increased values of $C_{1-0.2\;m,\;u}$ relate to increased values of *Q*_max_ except for VAD 131. This crossing panel was calibrated to a very soft and voided ballast support condition. Therefore, it cannot be claimed that an increased $C_{1-0.2\;m,\;u}$ will correspond to an increase in *Q*_max_ for all conditions. However, some level of increasing trend can be expected. The correlations between *Q*_max_ and $C_{1-0.2\;m,\;v}$ and $C_{1-0.2\;m,\;a}$ are stronger. Compared to Figure 16 (which is analogous to Figure 23a–c but with the same nominal track parameters for all crossing panels), the variability in the responses is now greater due to the variability in track properties. Even though the correlation is now worse at face value, the calibration shows the potential to increase the accuracy in the identification of the crossing geometry as the influence of the local track parameters on $C_{1-0.2\;m,\;\gamma }$ has now been partially accounted for. By scaling $C_{1-0.2\;m,\;\gamma }$ based on the local track conditions, a more fair comparison of crossing geometry status can be made between turnouts. However, it should be emphasized that a moderate proportionality still holds at the observed level of variation in track properties and that one can assume that a higher value for $C_{1-0.2\;m,\gamma }$ corresponds to a higher contact force and a more degraded crossing geometry.

Based on the presented results, it is difficult to conclude whether it is best to use $C_{1-0.2\;m,\;\gamma }$ computed from displacements, velocities, or accelerations. The velocity- and acceleration-based condition indicators show the best correlation with *Q*_max_ in simulations, suggesting that they are the better choice. By observing the mean values from Figure 22, no distinct trend is found that puts forward one version of the indicator as better than the other, in terms of the agreement between measurements and simulations. The discrepancies indicate that in situ parameters that are not accounted for in simulations, such as rail geometry outside of the scanning region, track irregularities, or check rail position, can have a significant influence on the results. Further investigations are needed to quantify the expected accuracy for the different condition indicators during operational condition monitoring.

## 8. Conclusions

This paper has presented key method developments for model-based condition monitoring of crossing panels. The studies were built around concurrently scanned crossing rail geometries and measured sleeper accelerations from six crossing panels in situ. The scanned geometries and a structural representation of the crossing panel were implemented in a multi-body simulation tool, from which the wheel–rail contact forces and corresponding dynamic track response were quantified for a sample of measured wheel profiles. The main areas of investigation included wheel–crossing interaction kinematics, calibration of track models to measured track responses, and a proposed crossing geometry condition indicator. Conclusions for these areas are elaborated below.

### 8.1. Wheel–Crossing Interaction Kinematics

From the multi-body simulation (MBS) parameter study with nominal track parameters, the influence of crossing geometry degradation on wheel–crossing interaction kinematics was computed using 19 different wheel profiles, three of which were hollow-worn. The transition locations between the wing rail and crossing nose and the corresponding dip angles were used as kinematic condition indicators. Different trajectory shapes based on the relative vertical wheel–rail displacement were observed between the crossings. For each crossing, a moderate variability was observed for the regularly worn profiles, while the hollow-worn wheels demonstrated outlying behavior. The transition locations from wing rail to crossing nose relative to the tip of the crossing nose were compared. For early and late transitions, higher maxima of the vertical wheel–rail contact forces were obtained. It was also observed that crossing panel VAD 131 had late and localized transition locations with higher wheel–rail impact forces, which is associated with more damage. Dip angles were computed for each crossing and wheel profile combination. A mostly linear trend between dip angle and maximum wheel–rail contact force was, as expected, found for all six crossings. It was shown that crossing panel VAD 131 was an outlier in terms of the extracted kinematic state indicators (higher impact angles, more localized transitions), and, thus, it was concluded that VAD 131 was in a highly degraded geometry state. Compared to a crossing panel with nominal rail geometry, the other crossing panels showed a small to moderate difference in their kinematic wheel–rail interaction.

### 8.2. Calibration of MBS Models

The calibration of MBS models for the six investigated crossing panels was based on several recordings of measured sleeper displacement. The calibration scheme included the tuning of linear or bi-linear (sleeper void model) ballast properties and rail pad properties. The results showed good to excellent agreement between measured and simulated sleeper displacements. Additionally, the measured and simulated sleeper velocities and accelerations showed a good to moderate fit. Overall, the calibrated parameters showed the potential to increase the accuracy in the identification of the crossing geometry. This was observed to be particularly important for the VAD 131 crossing case, where the difference between nominal and calibrated parameters and dynamic responses was the largest. As this was the severely degraded crossing case, the calibration successfully separated it from the group and emphasized its degraded condition. It should also be noted that the VAD 131 crossing was replaced 4 months after the crossing geometry scans. Considering this, it can be concluded that the observed state of VAD 131 indicates the need for crossing replacement.

Further, it was concluded that for more precise calibration of an MBS model based on measured sleeper accelerations, longer crossing rail geometry scans would be required together with track irregularity data. The current scans were from 1.8 to 2.7 m in length and it was observed that the influence of wheel positions outside that region influenced the sleeper acceleration response significantly.

Now that the potential of the model calibration has been demonstrated, the next step is to develop a fully automated calibration procedure using an optimization algorithm that can be used to robustly identify the crossing panel state. In the development of this algorithm, the inverse crossing geometry problem would be addressed, alleviating the need for crossing geometry scans and making the method more widely applicable for condition monitoring. Ultimately, the objective is to incorporate the model calibration routine in a Digital Twin for crossing panels that can not only monitor the condition using calibration but also simulate the future damage evolution and predict the next maintenance occasion. 

### 8.3. Crossing Geometry Condition Indicator

A crossing geometry condition indicator $C_{\lambda_{1}-\lambda_{2},\;\gamma }$ was proposed that is computed as the root mean square (RMS) of a sleeper response signal in a given band-pass region. For the present study, the indicator was computed for accelerations and reconstructed velocities and displacements. It was shown in simulations with nominal track parameters and the six scanned crossing geometries that this condition indicator has a strong correlation to the maximum wheel–rail contact force and, thus, the geometry condition of the crossing. The correlation between the crossing condition indicator and the maximum wheel–rail contact force was stronger than those that could be observed between the corresponding peak values of displacement, velocity, or acceleration and the maximum wheel–rail contact force. The condition indicators were computed and compared for measured and simulated track responses for the six crossing panels. The qualitative agreement between them suggests that the indicator can be used to qualitatively assess crossing geometry condition. A slightly better agreement was obtained for calibrated track parameters in the simulations. The discrepancies between indicator values in measurement and simulation indicate that in situ parameters that are not accounted for, such as rail geometry outside of the scanning region, track irregularities, or check rail position, can have a significant influence on the results. Future studies would be needed to more precisely address the accuracy that can be expected from the given method in operational condition monitoring.

The next steps in the development of the condition indicator are to (1) use repeated crossing geometry and acceleration measurements for the same in situ crossings to verify that it can detect a change in crossing geometry over time and to quantify the effect for a certain geometry change, (2) to account for the local track conditions, either via model calibration or in a simplified manner to make implementation easier, and (3) to suggest maintenance limits.

## Figures and Tables

**Table 1:** Crossing panel data.

Location	Höör	Höör	Stehag	Stehag	Vätteryd	Vätteryd
Crossing name	21B	22A	21A	21B	102	131
Design	EV-60E 1:18.5	EV-60E 1:18.5	UIC60 1:18.5	UIC60 1:18.5	UIC60 1:18.5	UIC60 1:18.5
Direction	Trailing	Facing	Facing	Facing	Trailing	Facing
Radius	1200 m	1200 m	1200 m	1200 m	1200 m	1200 m
Crossing rail installation date	2014	2014	2018	2012	2014	2014
Scanning date	4 June 2019					

**Table 2:** The 3D scanner properties.

Scanner Information	
Device	PorTable 3D laser scanner
Accuracy	0.035 mm
Volumetric accuracy	0.02 mm + 0.06 mm/m
Measurement resolution	0.025 mm
Mesh resolution	0.1 mm
Measurement rate	800,000 measurements/s
Light source	7 laser crosses
Scanning area	310 × 350 mm

**Table 3:** Konux GmbH accelerometer properties.

Device	Monoaxial Cellular Accelerometer
Installation	Permanent sleeper connection
Direction of measurements	Vertical
Sampling rate	2 kHz
Range	±50 g or ±100 g Depending on crossing panel

**Table 4:** Nominal MBS parameter values with vehicle parameters that correspond to an X2 train cabin car bogie.

MBS Model Components		Value
Vehicle	Type	Single bogie [28]
	Wheel radius [m]	0.46
	Wheelset mass [kg]	1340
	Axle load [kg]	15,500
	Axle spacing [m]	2.9
Rail *	Element type	Timoshenko beam
	Lengthwise node spacing [m]	0.3
	Profile	60E1
	Young’s modulus [GPa]	210
	Mass density [kg/meter rail]	60
Rail pads	Element type	Kelvin bushing elements
	Vertical stiffness [MN/m]	1200 (UIC60) 60 (EV-60E)
	Vertical damping [kNs/m]	25 (UIC60) 6 (EV-60E)
Sleeper	Element type	Timoshenko beam
	Lengthwise node spacing [m]	0.25 average
	Young’s modulus [GPa]	30
	Mass density [kg/m3]	2400
Ballast	Element type	Kelvin bushing elements
	Vertical stiffness [MN/m/m]	30
	Vertical damping [kNs/m/m]	125

* The equivalent cross section of crossing nose and wing rails is modeled as having three times the bending stiffness and mass of the standard UIC60 rail.

**Table 5:** Summary of operational conditions studied in the simulations.

Feature Name	Case 1—Nominal	Case 2—Soft Pad	Case 3—Calibration
Wheel profiles	19	19	19
Crossing panels	6 scanned + 1 nominal (7)	6 scanned + 1 nominal (7)	6 scanned
Train speed [km/h]	160	160	various
Total number of simulations	133	133	114

**Table 6:** Summary of calibrated track model parameters.

Calibration Parameters									
Crossing Panel	Ballast Vertical Stiffness [MN/m/m]	Ballast Vertical Damping [kNs/m/m]	Rail Pad Vertical Stiffness [MN/m]	Rail Pad Vertical Damping [kNs/m]	Number of Voided Sleepers	Sleeper Void [mm]	Crossing and Stock Rail Flexural Stiffness	RMS Error Measurem-ents and Simulations	Train Speed [km/h]
HO 21B	15	75	50	12	0	0	100%	0.037	160
HO 22A	20	60	60	12	2	0.55	100%	0.052	190
SG 21A	20	50	1200	120	3	1.00	100%	0.085	160
SG 21B	20	50	1200	120	3	2.00	100%	0.149	160
VAD 102	12	50	1200	120	0	0	100%	0.138	190
VAD 131	10	50	1200	120	4	5.00	75–85%	0.358	130

## Data Availability

Not applicable.
